# Changes in the spatial distribution of COVID-19 incidence in Italy using GIS-based maps

**DOI:** 10.1186/s12941-020-00373-z

**Published:** 2020-07-18

**Authors:** Cecilia Acuti Martellucci, Ranjit Sah, Ali A. Rabaan, Kuldeep Dhama, Cristina Casalone, Kovy Arteaga-Livias, Toyoaki Sawano, Akihiko Ozaki, Divya Bhandari, Asaka Higuchi, Yasuhiro Kotera, Zareena Fathah, Namrata Roy, Mohammed Ateeq Ur Rahman, Tetsuya Tanimoto, Alfonso J. Rodriguez-Morales

**Affiliations:** 1 Section of Hygiene and Preventive Medicine, Department of Biomedical Sciences and Public Health, University of the Marche Region, Ancona, Italy; 2 Department of Global Health Policy, School of International Health, Graduate School of Medicine, The University of Tokyo, Bunkyo-ku, Tokyo Japan; 3 Department of Microbiology, Tribhuvan University Teaching Hospital, Institute of Medicine, Kathmandu, Nepal; 4 Molecular Diagnostic Laboratory, Johns Hopkins Aramco Healthcare, Dhahran, Saudi Arabia; 5 Division of Pathology, ICAR-Indian Veterinary Research Institute, Izatnagar, Bareilly, Uttar Pradesh 243 122 India; 6 Istituto Zooprofilattico Sperimentale del Piemonte, Liguria e Valle d’Aosta (IZSPLV), Turin, Italy; 7 Faculty of Medicine, Universidad Nacional Hermilio Valdizán, Huánuco, Peru; 8 Masters in Clinical Epidemiology and Biostatistics, Universidad Cientifica del Sur, Calle Cantuarias 398, Miraflores, 15074 Lima, Peru; 9 Department of Surgery, Sendai City Medical Center, Sendai, Miyagi Japan; 10 Department of Public Health, Fukushima Medical University School of Medicine, Fukushima, Japan; 11 Department of Breast Surgery, Jyoban Hospital of Tokiwa Foundation, Iwaki, Fukushima Japan; 12 Medical Governance Research Institute, Minato-ku, Tokyo Japan; 13 University of Derby, Derby, UK; 14 Royal Holloway, University of London, London, UK; 15 SRM Medical College, Hospital and Research Center, Chennai, India; 16 Karnatak University, Dharwad, Karnataka India; 17 Public Health and Infection Research Group, Faculty of Health Sciences, Universidad Tecnologica de Pereira, Pereira, Risaralda Colombia; 18 Grupo de Investigación Biomedicina, Faculty of Medicine, Fundación Universitaria Autónoma de las Américas, Pereira, Risaralda Colombia

**Keywords:** SARS-CoV-2, COVID-19, Geographical information systems, Pandemic, Italy

Dear editor

Massive spreading of the pandemic Coronavirus Disease 2019 (COVID-19) in different continents [1, 2], have been observed [3]. Analyses mostly focused on the number of cases per country and administrative levels, multiple times without considering the relevance of the incidence rates. These help to see the concentration of disease among the population in terms of cases per 100,000 inhabitants. Even more, using geographical information systems (GIS)-based maps, stakeholders may rapidly analyze changes in the epidemiological situation [4–7]. Although the epidemic of COVID-19 caused by the severe acute respiratory syndrome coronavirus 2 (SARS-CoV-2) started in Italy on January 31, 2020, no reports on the use of GIS-based maps have been published to analyze the distinct differences in incidence rates across its regions and provinces during the last months. For these reasons, we have developed epidemiological maps of incidence rates using official populations, by regions (1st administrative level of the country) and provinces (2nd administrative level), for COVID-19 in Italy using GIS.

Surveillance cases data of the cumulative number at March 15, April 18, and June 8, 2020, officially reported by the Italian health authorities were used to estimate the cumulated incidence rates on those dates using reference population data on SARS-CoV-2 confirmed infections (cases/100,000 pop) and to develop the maps by regions and provinces, using the GIS software Kosmo^®^ 3.1, as performed in previous related studies [6, 7]. Starting on March 8, 2020, the region of Lombardy, together with 14 additional northern and central provinces, in Piedmont, Emilia-Romagna, Veneto, and Marche, they were put under lockdown. On March 10, 2020, the government extended the lockdown measures to the whole country.

Up to March 15, 2020, after 44 days of epidemics, 24,053 cases of COVID-19 were reported in the country, for a cumulated rate of 39.6 cases/100,000 population, reaching 174,103 cases during April 18, 2020, for a rate of 286.78, and 232,855 cases during June 8, 2020, for a rate of 383.56. All the regions of the country have been affected, with rates ranging from 59.42 (Calabria) to 947.75 cases/100,000 population (Aosta Valley/Vallée d’Aoste) (June 8, 2020) (Fig. 1). Higher diversity is found in provinces, where incidence rates ranged from 28.23 (Sud Sardegna, Sardinia) to 1811.37 (Cremona, Lombardy) (June 8, 2020) (Table 1). At Lombardy are located five of the top ten provinces with higher incidence rates (Table 1, Fig. 1), with considerable increases and changes from March 15, 2020, to June 8, 2020, in approximately 3 months (Fig. 1, Table 1). Cremona (Lombardy), Piacenza (Emilia-Romagna), and Lodi (Lombardy) have become in the geographic core of the cumulated incidence rate of COVID-19 in the north of the country and Italy (Fig. 1).

**Fig. 1:**
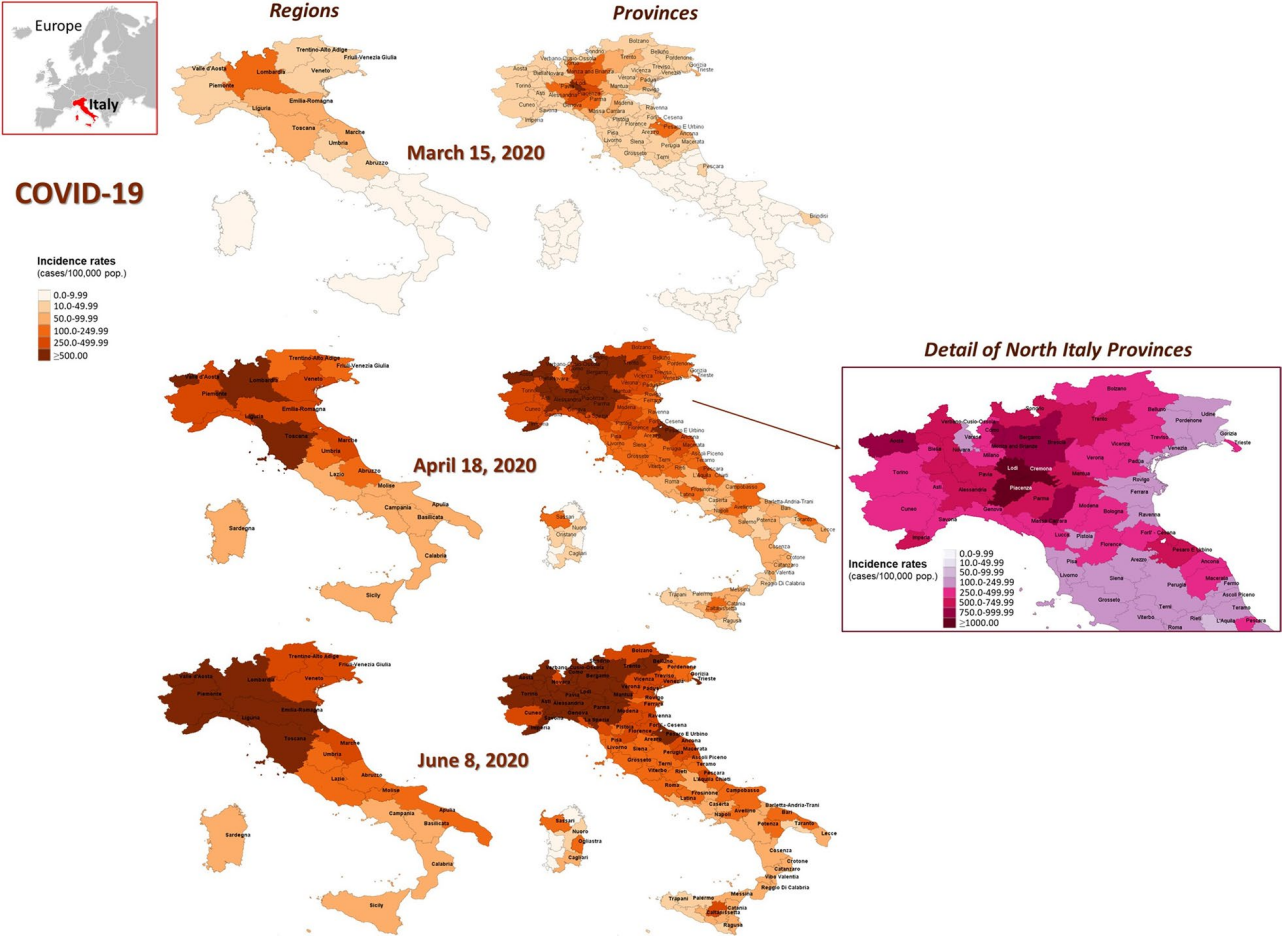
COVID-19 situation in Italy, on March 15, April 18, and June 8, 2020, by regions and provinces

**Table 1:** Top ten provinces by incidence rate (cases/100,000 inhabitants), of COVID-19, Italy, on March 15, April 18, and June 8, 2020

Region	Province	Population^a^	Cases^b^			Incidence rates^c^		
			15-Mar	18-Apr	8-Jun	15-Mar	18-Apr	8-Jun
Lombardy	Cremona	358,955	1792	5407	6502	499.23	1506.32	1811.37
Emilia-Romagna	Piacenza	287,152	1012	3299	4506	352.43	1148.87	1569.20
Lombardy	Lodi	230,198	1320	2714	3502	573.42	1178.99	1521.30
Lombardy	Bergamo	1,114,590	3416	10,629	13,609	306.48	953.62	1220.99
Lombardy	Brescia	1,265,954	2473	11,758	15,070	195.35	928.79	1190.41
Lombardy	Pavia	545,888	722	3536	5418	132.26	647.75	992.51
Aosta Valley/Vallée d’Aoste	Aosta Valley/Vallée d’Aoste	125,666	57	1073	1191	45.36	853.85	947.75
Piedmont	Alessandria	421,284	207	2752	3968	49.14	653.24	941.88
Emilia-Romagna	Reggio nell’Emilia	531,891	185	4217	4962	34.78	792.83	932.90
Piedmont	Asti	214,638	87	1038	1857	40.53	483.60	865.18

^a^From ISTAT. “Italian National Institute of Statistics—Resident Population.” Demographic Statistics. Accessible at http://demo.istat.it/. Accessed on 8 June 2020

^b^Italian Civil Protection Department [COVID-19 Italy—Situation monitoring]. Accessible at http://opendatadpc.maps.arcgis.com/apps/opsdashboard/index.html#/b0c68bce2cce478eaac82fe38d4138b1. Accessed on 8 June 2020

^c^Cases per 100,000 inhabitants

From the GIS-based maps, it is clear that spreading in the country is occurring from north regions and provinces such as Lombardy. On March 15, 2020, most of the southern regions were not affected (Fig. 1), but approximately a month later, all of them reported COVID-19 cases (Fig. 1), including the insular regions of Sicily and Sardinia. While the change between April 18 and June 8, has been 33%, there is still a concern in the country, mainly because, in this time, the number of deaths has reached over 34,000 deaths (14.6%).

Italy reached the top of countries with the highest number of reported COVID-19 cases, now is the ninth country in cumulated cases. It is the fourth in the European region, after Russia, the United Kingdom, and Spain. Italy represented one of the most significant sources of imported cases for other continents, as is the case of Latin America, that received their first cases from Milan, Lombardy [8–10].

Patient 1 of Italy (it was not possible to find patient 0) was discovered on February 20, 2020, when a 38-year-old man from the city of Codogno had shown up at the hospital. Since that date, two large clusters of outbreaks have spread first in Northern Italy, later all over the country (Fig. 1) [11]. Cases are multiplying, and the national healthcare system is collapsing [12–14]. Many regions are increasing intensive care beds, revolutionizing entire hospital wards. In Italy, the coordination of the swabs is managed regionally. Once the epidemic began, for example, the Veneto region started immediately with active surveillance, i.e., on asymptomatic, and this contained the spread of the virus compared to other Northern Regions.

The health system is indeed regionalized, and dispositions of the Ministry of Health are translated into multiple regional decrees and regulations, often at different timings [15]. Many regions, for example, adopted evolving criteria for testing and diagnosis, according to dispositions from the central Government, but also to test capacity, which was heavily reliant on the availability of reagents. Samples were collected either in healthcare facilities, in provisional collection points, or even in people’s houses, depending on the region and the phase of the pandemic. The epitome of these differences is the two most heavily affected regions, Lombardy and Veneto. Lombardy hospitalized, even cases with relatively modest symptoms, causing numerous nosocomial outbreaks (9% of infections were among health professionals until March). Veneto, instead, deployed widespread testing since the beginning, maintaining disease management as much as possible at the primary health care level [15].

The healthcare workers are facing COVID-19 pulling 12 h shifts in critical situations with minimal to non-existent personal protective equipment (PPE) [12–14]. Lacking PPE led both many healthcare workers to become COVID-19 positive (7145), and to the death of several doctors (51; about 9% of the total cases; March 27, 2020) [11].

As observed in the GIS-based maps, the COVID-19 spreading in the country has been significant and moving from north to south across the time, with provinces reaching more than 1000 cases per 100,000 inhabitants (Fig. 1) [12–14]. Differences in the incidence by regions would be related to different social and economic factors. Such as people who travel abroad, for whom there is a sharp difference between northern regions (about 26% of travelers) and central and southern regions (about 19%). Or net income at the household level, which is ranging from 35,000€ in the North-east to 26,000€ in the South [16]. Additionally, as has been recently suggested, climatic conditions could also influence the transmission of SARS-CoV-2 [17].

Testing capacity increased over time. While some degree of ascertainment bias is inevitable, the number of swabs performed nationally stabilized around 70,000 per day in mid-April when the peak in the number of active cases was registered (Fig. 1). From that point onwards, daily cases only decreased, consistently with the impact of the lockdown imposed in early March. Besides, and most importantly, while it is true that the epidemic might have gone undetected for some time before Case 1 was discovered in Codogno, the growth in ICU beds demand for subjects with respiratory failure that ensued in the following weeks is most likely explained by a substantial increase of cases.

Considering the limitations of diagnostics and the asymptomatic cases, these figures would be many times more. Further characterization studies should include multiple GIS-based maps with other variables at the regions and provinces levels such as deaths, hospitalizations, and ICU rates per population to understand better the critical situation of the country and its administrative levels.

## Data Availability

If required.
